# Methods and Initial Data for A Massive Real‐World Deeply Phenotyped Cohort Study

**DOI:** 10.1002/alz.092172

**Published:** 2025-01-09

**Authors:** Craig W Ritchie, John E Harrison, Miles Welstead, Joseph Milne, Alison Green, Clara Doran, Zain Hussain, Laura Kaminioti‐Dumont, Andrew Lan, Ayeda Nadeem, Tara Noonan, Azin Poon, Kristiina Rannikmäe, Tanya van der Westhuizen, Sarah Gregory

**Affiliations:** ^1^ Scottish Brain Sciences, Edinburgh United Kingdom; ^2^ King’s College ‐ Institute of Psychiatry, Psychology & Neuroscience, London United Kingdom; ^3^ Scottish Brain Sciences, Edinburgh, Scotland United Kingdom; ^4^ Alzheimer Center Amsterdam, Amsterdam UMC, Vrije Universiteit Amsterdam, Amsterdam Netherlands; ^5^ Scottish Brain Sciences, Edinburgh, Lothian United Kingdom; ^6^ University of Glasgow, Glasgow, Glasgow United Kingdom; ^7^ NHS Forth Valley, Forth Valley, Forth Valley United Kingdom; ^8^ University of Edinburgh, Edinburgh United Kingdom

## Abstract

**Background:**

Recent therapeutic successes in the treatment of Alzheimer’s disease (AD), and diagnostic tools such as blood‐based biomarkers, have energised the field to develop more specific diagnostics and therapies following the best principles of precision medicine. Large, ‘real‐world’ cohorts are needed to understand whether these, and other future breakthroughs, are valid when employed in the wider AD community.

**Methods:**

Our cohort study will provide a well‐phenotyped (combining data on risk factors for neurodegenerative disease, cognitive assessment results, disease biomarkers and genetics) population aged 50 and above across the spectrum of risk for, and the earliest disease stages of, neurodegeneration (beginning in Scotland). Participants complete an innovative cognitive testing battery (combining elements of the ACE‐R (including MMSE), TICS, RBANS and digital cognitive tests), assessments of global functioning, questionnaires about risk factors for neurodegenerative disease, physical health assessments and collection of blood samples for clinical blood tests and storage for future research. A sub‐set of participants are invited to join the Enhanced Phenotyping cohort and additionally undergo brain MRIs and lumbar punctures. Cerebrospinal fluid will be analysed for amyloid beta‐42, phosphorylated tau‐181 and total tau, with additional aliquots stored for future research use. Participants will be followed up every 6 months to complete questionnaires, with more comprehensive visits annually. A minimum of 3000 participants will be recruited to the Enhanced Phenotype cohort with an estimated 7,000 in the cohort overall.

**Results:**

A total of 11 participants have been recruited to the cohort to date (mean age: 69 years; 6 (55%) female). Mean ACE‐R and MMSE scores are 92 and 28 respectively. Data collection is ongoing with recruitment anticipated to continue throughout 2024 and 2025, with a minimum of 100 participants recruited by July 2024. Baseline data, including AD biomarker status where available, will be presented at AAIC 2024.

**Conclusion:**

This an ambitious project aiming to recruit a deeply phenotyped, real‐world representative cohort of participants across the spectrum of risk for, and in the earliest stages of, neurodegenerative conditions. The data and samples collected as part of this cohort will be invaluable to better understanding disease trajectories, and developing and testing new diagnostics and therapeutics.

